# Peer-Tutoring as a Student-Centered pedagogy in Nursing research Education: Effects on Knowledge Acquisition and Academic Self-Confidence

**DOI:** 10.12688/f1000research.179096.1

**Published:** 2026-04-17

**Authors:** Jansi Rani Natarajan, Thilagavathi Krishnasamy, Reshma Prakash Sreekala, Irene Dorothy Natarajan, Richard Mottershead, Vidya Seshan

**Affiliations:** 1 College of Health Sciences, University of Buraimi, Al Buraimi, Oman; 2 Indian School Bousher, Muscat, Oman; 3 University of Baghdad, Baghdad, Iraq; 4 University of Sharjah, Sharjah, United Arab Emirates

**Keywords:** Peer tutoring; Nursing education; Nursing research education; Academic self-confidence; Student-centered learning; Undergraduate nursing students; Oman

## Abstract

**Background:**

Nursing research education is crucial for developing competencies in evidence-based practice. However, undergraduate nursing students often perceive research courses as complex and abstract, negatively affecting learning outcomes and academic self-confidence. Peer tutoring, a student-centered pedagogical approach, may enhance engagement and learning in higher education. Yet empirical evidence on its effectiveness in teaching nursing research, particularly in the Gulf region, is limited.

The aim of this study evaluated the effectiveness of peer tutoring in improving knowledge acquisition and academic self-confidence among undergraduate nursing students enrolled in a nursing research course.

**Methods:**

A quasi-experimental post-test control group design was conducted at the College of Health Sciences, University of Buraimi, Oman, during Fall 2025. A total of 137 students participated: 70 in the experimental group and 67 in the control group. The experimental group received structured peer-tutoring sessions alongside traditional lectures, while the control group received lectures only. Data were collected using a demographic questionnaire, a faculty-developed knowledge assessment, an academic self-confidence scale, and a perceived usefulness of peer tutoring questionnaire for the experimental group. Independent samples t-tests and Pearson’s correlation analyses examined group differences and associations.

**Results:**

Students exposed to peer tutoring achieved significantly higher knowledge scores (p < .001) and academic self-confidence (p < .001) than the control group. The intervention had a moderate effect on knowledge and a large effect on academic self-confidence. Knowledge and academic self-confidence were positively correlated (r = .34, p < .001). Most participants reported high perceived usefulness of peer tutoring.

**Conclusion:**

Peer tutoring is an effective strategy for enhancing knowledge acquisition and academic self-confidence in nursing research education. Integrating structured peer-tutoring approaches into undergraduate nursing curricula may promote deeper learning and strengthen research competence among future nurses.

## Introduction

Nursing education has increasingly shifted toward student-centered, active learning pedagogies aimed at fostering high-order thinking, collaboration, and learner autonomy. Peer tutoring is a collaborative learning strategy in which students teach and support one another, fostering academic growth, confidence, and social skills for both tutors and tutees in a flexible, relatable environment particularly in theoretically demanding courses such as nursing research. As research competence is foundational to evidence-based nursing practice, identifying effective instructional strategies that enhance students’ engagement, understanding, and academic self-confidence remains a priority ( Al Yahyaei et al., 2024).

Peer tutoring is a collaborative learning strategy in which students support one another’s learning through structured or semi-structured academic interactions ( Kim et al., 2021). Grounded in social constructivist learning theory, peer tutoring emphasizes knowledge co-construction, reciprocal learning, and active participation. When learners assume teaching responsibilities or study alongside their classmates, they frequently demonstrate improvements in thinking, communication, and self-assurance. Research also indicates that peer learning may be equally effective as lectures in improving academic performance and equipping students for clinical practice ( Choi et al., 2021; Muthalib et al., 2023). An additional advantage of peer learning is its ability to reduce the loneliness experienced by contemporary students, promoting an environment of teamwork and mutual accountability in education ( Koo et al., 2024). Several investigations have linked this approach to improvements in achievement, analytical skills, and learner satisfaction ( Choi et al., 2021; Wang, 2024). Peer tutoring is a widely recognized, evidence-based instructional strategy employed across various disciplines. Despite its enduring popularity, comprehensive research on its effectiveness in teaching nursing research concepts remains scarce.

Evidence from nursing education literature indicates that peer tutoring contributes positively to students and non-students academic achievement, satisfaction, and sense of belonging ( Kim et al., 2021b; Mottershead, 2022). Students who participated in tutoring consistently performed better academically than those who did not and reported that the sessions were helpful to their learning ( Im Kang et al., 2021; Dias et al., 2026).

Despite the growing body of evidence supporting peer tutoring in clinical, skill-based, and foundational nursing courses, its application in nursing research education remains underexplored. Nursing research courses are often perceived as abstract, complex, and anxiety-provoking by undergraduate students, leading to reduced engagement and lower academic confidence. While innovative instructional approaches have been advocated to address these challenges, empirical evidence examining peer tutoring as a strategy for improving learning outcomes in nursing research courses is limited. This gap is particularly evident in the Gulf region, including Oman, where cultural norms favor collaborative learning and peer interaction, potentially enhancing the effectiveness of peer-assisted pedagogies.

Recent call in nursing education literature emphasize the need for innovative, evidence-based teaching strategies to strengthen research literacy and scholarly inquiry among undergraduate nursing students ( Catarelli et al., 2023; Dias et al., 2026). Integrating peer tutoring into nursing research education aligns with contemporary curricular reforms that prioritize competency-based education, collaborative practice, and lifelong learning skills. As nursing programs increasingly emphasize self-directed learning, collaborative practice, and development of scholarly inquiry capacities, peer tutoring provides a proven mechanism for cultivating these capabilities. However, rigorous evaluation of its effectiveness within this context is necessary to inform curriculum design and teaching practice.

To date, no published studies have systematically examined the impact of peer tutoring on learning outcomes in undergraduate nursing research courses in Oman or the wider Gulf region. Addressing the gap is critical, as understanding the pedagogical value of peer tutoring in research education may support the development of more engaging, inclusive, and effective teaching strategies. Such evidence is also aligned with national educational priorities emphasizing quality, innovation, and student-centered learning.

Therefore, the present study aimed to evaluate the effectiveness of peer tutoring in improving knowledge acquisition and academic self-confidence among undergraduate nursing students enrolled in Nursing Research course at a private university in Oman. By examining learning outcomes within this under-researched context, the study seeks to contribute empirical evidence to the nursing education literature and inform best practices for teaching research competencies in undergraduate nursing programs.

## Method

### Study design

A quasi-experimental post-test control group design was employed to examine the effectiveness of peer tutoring on learning outcomes among undergraduate students enrolled in a Nursing Research course. This design was selected to allow comparison between students exposed to the peer tutoring intervention and those receiving traditional instruction while maintaining feasibility within an academic setting.

### Setting

The study was conducted at the College of Health Sciences, University of Buraimi, Oman, during the Fall 2025 semester. The University of Buraimi is a private higher education institution comprising four colleges, including the College of Health Sciences. The Bachelor of Science in Nursing program enrolls approximately 1,200 students and requires completion of 135 credit hours over four academic years. The Nursing research course is a core component of the undergraduate curriculum, focusing on foundational research concepts, and group-based proposal development.

### Participants and sampling

Participants were undergraduate nursing students enrolled in the Nursing Research course during the Fall 2025 semester. Approximately 200 students were registered across morning and evening sections of the course. A convenience sampling technique was used to recruit participants from both sections.

### Sample size

Sample size estimation was conducted using G*Power software for a two-group comparison, assuming a moderate effect size (ƒ2 = 0.30), a significance level of alpha = 0.05, and a statistical power of 0.80. The required minimum sample size was determined to be 70 Students, distributed between the experimental and control groups.

### Inclusion and exclusion criteria

Inclusion criteria comprised enrollment in the nursing research course during the fall 2025 semester and willingness to participate in the study. Students who were absent during scheduled peer-tutoring sessions were excluded from data collection.

### Intervention: peer tutoring program

The Nursing Research course spanned 15 weeks, including course orientation in week 1, a midterm examination in week 8, and the final examination in week 15. Peer tutoring sessions were implemented during weeks 7, 9, 10, and 11 of the semesters. An open invitation to serve as a peer tutor was extended to the students in the experimental group. Interest Students volunteered to deliver brief micro-teaching sessions of approximately 10 minutes on assigned research topics aligned with the course objectives.

Faculty members evaluated volunteer tutors using a standardized microteaching Criteria Checklist assessing content accuracy, clarity, organization, engagement strategies, questioning techniques, and facilitation skills. Fourteen peer tutors were selected, maintaining an approximate tutor-to-tutee ratio of 1:4–5. Selected peer tutors participated in three brief preparatory sessions focused on reinforcing key research concepts, effective small group facilitation, and constructive feedback provision. Tutors were provided with structured teaching materials including case analyses, worksheets, guided questions, and practice exercises.

Both experimental and control group received standard faculty-led lectures covering the same Nursing Research content. I addition, students in the experimental (morning) session engaged in peer tutoring activities during scheduled course support time, with minimal faculty oversight to ensure consistency across sessions. Students in the control (evening) session received only traditional lecture-based instruction.

At the conclusion of the intervention period, Students in the experimental group completed self-report questionnaires assessing knowledge, academic self-confidence, and perception of the usefulness of peer tutoring. Students in the control group completed a self-reported questionnaire and academic self-confidence only.

### Data collection instruments

#### Demographic Questionnaire

Participants’ demographic characteristics were collected using a structured questionnaire, including age, sex, cumulative grade point average (GPA), and residential status.

#### Knowledge Assessment

Knowledge of nursing research concepts was measured using 10 faculty-developed multiple-choice questions (MCQs) aligned with course content. Items were formatted in NCLEX-style and mapped to three cognitive levels: comprehension, application, and higher-order thinking. The MCQs were developed by the course coordinator and reviewed by external subject-matter experts to ensure content validity and relevance.

#### Academic Self-Confidence Scale

Academic self-confidence was measured using a revised version of the Self-Confidence Scale originally developed by Sander and Sanders (2003). The instrument comprises 14 items rated on a 5-point Likert scale ranging from 1 (No confidence at all) to 5 (Complete confidence). Higher total scores indicate greater academic self-confidence.

#### Perceived Usefulness of Peer Tutoring

Students in the experimental group completed the Usefulness of Peer Tutoring Scale developed by Thomson, Smith, and Annesley (2014). The scale consists of 18 items rated on a 5-point Likert scale (1 = Strongly disagree to 5 = Strongly agree) and demonstrates strong construct validity and internal consistency (Cronbach’s α = 0.80) ( Thomson et al., 2014). Higher scores reflect more positive perceptions of peer tutoring effectiveness.

#### Data Analysis

Data was analyzed using IBM SPSS Statistics version 30. Descriptive statistics, including means, standard deviations, frequencies, and percentages, were used to summarize participant characteristics and outcome variables. Independent samples t-tests were conducted to compare knowledge and academic self-confidence scores between the experimental and control groups. Pearson’s correlation coefficient was used to examine the relationship between knowledge scores and academic self-confidence. Statistical significance was set at p < .05.

### Ethical considerations

Ethical approval was obtained from the Research and Ethics Committee of College of Health Sciences, University of Buraimi, Oman prior to data collection. The Ethical approval number for this study is AY25-26COHS-010. Written informed consent was obtained from all participants. Participation was voluntary, and students were assured that their decision to participate or withdraw would not affect their academic standing or course grades. All data were anonymized and stored securely in password-protected electronic files and locked cabinets accessible only to the research team. Participant confidentiality was maintained throughout the study and in all subsequent reports and publications.

## Result

A total of 137 undergraduate nursing students participated in the study, comprising 70 students in the experimental group and 67 in the control group. Participant demographic characteristics are summarized in Table 1.

**Table 1.** Demographic characteristics of Participants in the experimental and control groups.

Demographic characteristics	Category	Experimental group		Control group		Chi-square/ANOVA
		Frequency	Percent	Frequency	Percent	
Gender	Male	8	5.8	8	5.8	0.926
	Female	62	45.3	59	43.1	
Residence status	Inside the campus	41	29.9	43	31.4	0.501
	Outside the campus	29	21.2	24	17.5	
Age of the participants		Mean = 25.14, SD = 6.575		Mean = 22.27, SD = 3.131		0.001
CGPA		Mean = 3.28, SD = 0.674		Mean = 2.91, SD = 0.599		0.001

The majority of participants in both groups were female. In the experimental group, 62 students (88.6%) were female and 8 (11.4%) were male, while in the control group, 59 students (88.1%) were female and 8 (11.9%) were male. No statistically significant difference was observed between groups with respect to gender distribution (χ ^2^ = 0.926, p > .05).

With regard to residence status, 41 students (58.6%) in the experimental group and 43 students (64.2%) in the control group resided on campus, whereas 29 students (41.4%) and 24 students (35.8%), respectively, lived off campus. This difference was not statistically significant (χ ^2^ = 0.501, p > .05).

However, statistically significant differences were observed between the two groups in age and cumulative grade point average (CGPA). The mean age of students in the experimental group was higher (M = 25.14 years, SD = 6.58) than that of the control group (M = 22.27 years, SD = 3.13), and this difference was statistically significant (p = .001). Similarly, the experimental group demonstrated a significantly higher mean CGPA (M = 3.28, SD = 0.67) compared with the control group (M = 2.91, SD = 0.60; p = .001).

### Comparison of knowledge and academic self-confidence between groups

Independent samples t-tests were conducted to examine differences in knowledge and academic self-confidence between students who participated in peer tutoring and those who received traditional instruction only **(** Table 2 **)**.

**Table 2.** Comparison of knowledge and academic self-confidence between experimental and control groups.

Experimental/control		N	Mean	Std. deviation	t value	P value
Knowledge of the participants	Experimental group	70	9.71	0.640	3.702 ^***^	0.001
	Control group	67	8.94	1.623		
Academic self-confidence of the participants	Experimental group	70	56.56	8.326	3.643 ^***^	0.001
	Control group	67	48.04	7.799		

### Knowledge

Students in the experimental group demonstrated significantly higher knowledge scores (M = 9.71, SD = 0.64) than those in the control group (M = 8.94, SD = 1.62). This difference was statistically significant, t(135) = 3.70, p < .001. The 95% confidence interval for the mean difference ranged from 0.36 to 1.19. Effect size analysis indicated a moderate effect of the peer tutoring intervention on knowledge outcomes (Cohen’s d = 0.63, 95% CI: 0.29–0.98).

### Academic self-confidence

Similarly, academic self-confidence scores were significantly higher among students in the experimental group (M = 56.56, SD = 8.33) compared with the control group (M = 48.04, SD = 7.80). This difference was statistically significant, t(135) = 3.64, p < .001, with a 95% confidence interval for the mean difference ranging from 5.78 to 11.24. The magnitude of the intervention effect on academic self-confidence was large (Cohen’s d = 1.05, 95% CI: 0.70–1.41).

### Perceived usefulness of peer tutoring

**Figure 1.**
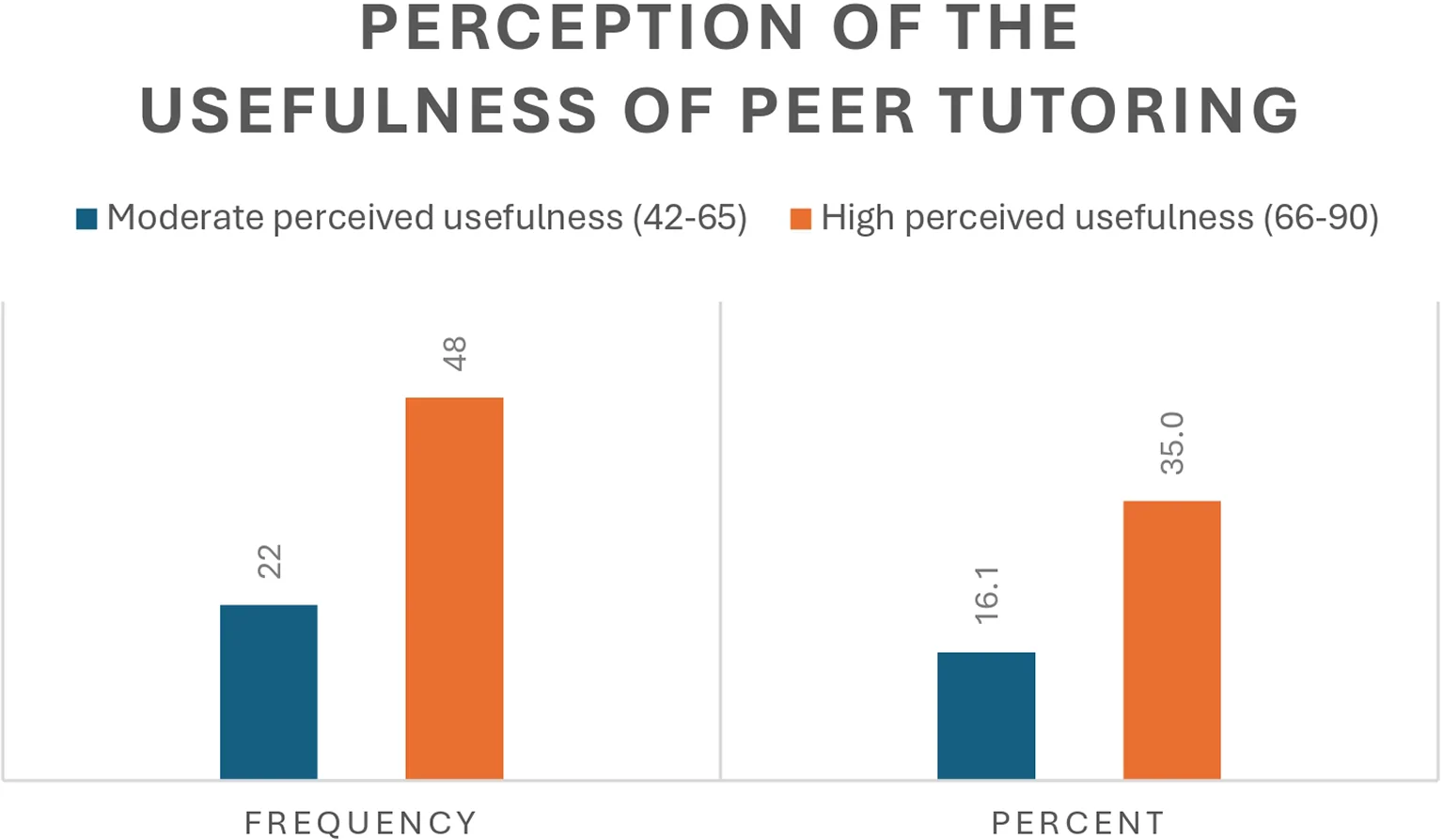
Perception of usefulness of Peer tutoring.

Perceptions of the usefulness of peer tutoring were assessed among students in the experimental group only. As illustrated in Figure 1, the majority of participants reported highly perceived usefulness of peer tutoring. Specifically, 48 students (68.6%) scored within the high perceived usefulness range (scores 66–90), while 22 students (31.4%) reported moderate perceived usefulness (scores 42–65). None of the participants reported low perceived usefulness. These findings indicate a consistently positive perception of peer tutoring among students exposed to the intervention.

### Relationship between knowledge and academic self-confidence

Pearson’s correlation analysis was performed to examine the relationship between knowledge and academic self-confidence among all participants (N = 137). A moderate, positive, and statistically significant correlation was identified between knowledge scores and academic self-confidence (r = .34, p < .001), indicating that higher levels of knowledge were associated with higher levels of academic self-confidence ( Table 3).

**Table 3.** Correlation between knowledge and academic self-confidence among Participants.

Variables	Mean	Std. Deviation		Knowledge of the participants	Academic self-confidence of the participants
Knowledge of the participants	9.34	1.279	Pearson Correlation		.342 ^***^
			Sig. (2-tailed)		0.000
Academic self-confidence of the participants	52.39	9.107	Pearson Correlation	.342 ^***^	
			Sig. (2-tailed)	0.000	

*** Correlation at 0.001 (2-tailed).

Additional analyses examining associations between demographic variables and outcome measures revealed that higher CGPA, older age, and off-campus residence were significantly associated with higher knowledge and academic self-confidence scores (p < .05).

## Discussion

The present study demonstrated that undergraduate nursing students who participated in peer tutoring within a nursing research course achieved significantly higher knowledge scores and academic self-confidence compared with those who received traditional lecture-based instruction. These findings are consistent with previous empirical studies conducted among nursing students in clinical skills and foundational courses in Oman and Turkey, which reported superior academic and affective outcomes following peer-assisted learning interventions ( Al Yahyaei et al., 2024; Şengül et al., 2022).

The observed improvement in knowledge acquisition supports existing evidence that peer tutoring facilitates deeper learning through interactive discussion, clarification of complex concepts, repeated engagement with content, and meaningful social interaction ( Al Yahyaei et al., 2024; Dias et al., 2025; Kim et al., 2021). In the context of nursing research education, where students are required to develop abstract reasoning and proposal-writing skills, peer tutoring may enable learners to process content more actively and contextualize research concepts through collaborative dialogue. Furthermore, observational learning, peer modeling, and social persuasion appear to contribute to improved academic self-confidence, particularly when students receive feedback in a supportive peer-led environment ( Feng et al., 2024; Mottershead & Alonaizi, 2022).

A notable contribution of this study is the significant enhancement of academic self-confidence among students who engaged in peer tutoring. Nursing research courses are frequently perceived by students as conceptually difficult and intimidating, often leading to reduced confidence and engagement. Peer tutoring offers a student-centered pedagogical approach that creates a psychologically safe learning space, enabling students to ask questions freely, articulate uncertainties, and receive peer affirmation. These findings align with earlier studies reporting increased psychological empowerment, academic self-efficacy, and confidence among nursing students participating in peer learning initiatives ( Al Yahyaei et al., 2024; Dias et al., 2025; Kim et al., 2021; Shaukat & Bashir, 2016).

The positive and statistically significant correlation between knowledge and academic self-confidence identified in this study further reinforces the interdependent relationship between cognitive and affective learning outcomes. As students’ understanding of nursing research concepts increased, their confidence in engaging with academic tasks also improved. This relationship is particularly salient in research education, where inadequate confidence can act as a barrier to active participation, critical inquiry, and scholarly engagement.

Students’ perceptions of the usefulness of peer tutoring further support the quantitative findings. The majority of participants in the experimental group rated peer tutoring as highly useful, reflecting strong acceptance of the intervention and perceived value in collaborative and individualized learning opportunities. This finding is consistent with prior research demonstrating high levels of student satisfaction and perceived effectiveness of peer tutoring in nursing education ( Kim et al., 2021; Lin et al., 2025; Wang, 2024). Within the Omani cultural context, which emphasizes collectivism and group-oriented learning, peer tutoring may be particularly effective in fostering motivation, engagement, and shared responsibility for learning.

Associations between higher CGPA, older age, off-campus residence, and improved knowledge and self-confidence scores may reflect greater academic maturity, self-directed learning skills, and prior educational exposure. Nevertheless, despite these baseline differences, the peer tutoring intervention was associated with significant improvements in learning outcomes, suggesting that the instructional strategy itself played a meaningful role in enhancing students’ academic performance and confidence.

Importantly, this study extends the existing body of nursing education literature by demonstrating the effectiveness of peer tutoring in a theoretical course, namely nursing research, rather than in clinical or skills-based settings alone. To the authors’ knowledge, this is among the first studies conducted in Oman to evaluate peer tutoring within a nursing research course. As such, the findings provide region-specific evidence supporting the integration of student-centered pedagogical strategies in Gulf nursing education and align with Oman Vision 2040, which emphasizes innovation, quality, and learner-centered approaches in higher education.

### Implications for nursing education and practice

The findings of this study have important implications for nursing education, curriculum design, and faculty development. The demonstrated effectiveness of peer tutoring in enhancing knowledge acquisition and academic self-confidence suggests that this student-centered pedagogical approach can be strategically integrated into undergraduate nursing curricula, particularly in theoretically demanding courses such as nursing research. Incorporating structured peer tutoring may help address students’ learning anxiety, improve engagement, and foster deeper conceptual understanding, thereby strengthening research literacy and evidence-based practice competencies among future nurses.

From a curricular perspective, peer tutoring aligns well with contemporary competency-based and learner-centered educational frameworks that emphasize collaboration, self-directed learning, and critical inquiry. Embedding peer tutoring within nursing research courses may support the development of transferable skills such as communication, teamwork, and scholarly thinking, which are essential for professional nursing practice. Additionally, the reciprocal benefits observed among peer tutors highlight the potential of peer-assisted learning to promote leadership development and metacognitive growth among high-performing students.

At the institutional level, the adoption of peer tutoring programs represents a cost-effective and sustainable strategy to enhance academic support without increasing faculty workload. Faculty members play a critical role in designing, monitoring, and evaluating peer tutoring interventions; therefore, targeted faculty development initiatives are recommended to ensure effective implementation and quality assurance. In the context of Oman and the wider Gulf region, where collaborative learning is culturally congruent, peer tutoring may be particularly effective in promoting inclusive and supportive learning environments. The study’s findings also support national higher education priorities focused on innovation, quality improvement, and student engagement.

### Limitations

Several limitations should be considered when interpreting the findings. First, the quasi-experimental design and use of convenience sampling limit causal inference and may introduce selection bias. Baseline differences in age and CGPA between the experimental and control groups may have influenced outcomes, although statistical analyses were conducted to account for these differences. Second, academic self-confidence and perceived usefulness were assessed using self-reported instruments, which may be subject to response and social desirability bias. Finally, the study was conducted at a single private university, which may limit the generalizability of the findings to other institutions or public universities in Oman.

### Recommendations

Based on the findings, several recommendations are proposed. Nursing educators should consider integrating structured peer tutoring into nursing research courses to enhance students’ understanding and academic self-confidence. Peer tutoring may also be extended to other theory-based nursing courses, such as statistics, ethics, and leadership, where students commonly encounter learning challenges. Faculty development programs should include training on the design, implementation, and evaluation of peer tutoring interventions to ensure instructional consistency and effectiveness. Future research should employ randomized controlled designs and multi-institutional samples to strengthen generalizability. Longitudinal studies are recommended to examine the sustained impact of peer tutoring on knowledge retention and research competence, while qualitative investigations could provide deeper insight into students’ and tutors’ lived experiences of peer-assisted learning.

## Conclusion

This study provides evidence that peer tutoring is an effective instructional strategy for enhancing both knowledge and academic self-confidence among undergraduate nursing students enrolled in a nursing research course. Students who participated in peer tutoring demonstrated superior learning outcomes compared with those who received traditional instruction and reported high perceived usefulness of the approach. Incorporating peer tutoring into nursing education curricula may contribute to the development of more confident, competent, and research-literate future nurses.

## Data Availability

Participant data contains sensitive personal information, and sharing such data publicly could compromise confidentiality and anonymity. The Institutional Review Board (IRB) has mandated that data sharing is permissible only under specific conditions that ensure participant privacy and align with ethical guidelines. Access to the data may be granted to qualified researchers for legitimate academic purposes upon request. Requests for access must be submitted in writing to the corresponding author, Dr. Richard Mottershead, richmottershead@gmail.com.
