# Supplementary material for: Peer-Tutoring as a Student-Centered pedagogy in Nursing research Education: Effects on Knowledge Acquisition and Academic Self-Confidence
Source: F1000Res. 2026 Jul 1;15:546. Originally published 2026 Apr 17. [Version 2] doi: 10.12688/f1000research.179096.2 (PMC13550914; doi:10.12688/f1000research.179096.2)
Supplement: Peer review history

# Reviewer report: Reviewer response for version 2

**DOI:** 10.5256/f1000research.203296.r499890

**Published:** 2026-07-13

**Authors:** Saeeda Khan, Yasir Ali

**Affiliations:** 1 Ziauddin University, Karachi, Sindh, Pakistan; 2 Nursing Education, Ziauddin University Faculty of Nursing & Midwifery, Karachi, Sindh, Pakistan

This study is a good and relevant contribution to nursing education, as it addresses the common challenge of students finding research courses complex and abstract. The use of a quasi-experimental design with a reasonable sample of 137 students and strong statistical results showing significant improvements in both knowledge and academic self-confidence supports the effectiveness of peer tutoring. The high perceived usefulness also indicates good acceptability among students.

 However, the study has some limitations including the lack of a pre-test, use of a single site, and limited details on the peer tutor training and intervention process, which affect generalizability. Overall, it is a promising study that provides practical evidence for integrating peer tutoring into nursing research courses, but further multi-site research with stronger design is recommended.

Is the work clearly and accurately presented and does it cite the current literature?

Yes

If applicable, is the statistical analysis and its interpretation appropriate?

Yes

Are all the source data underlying the results available to ensure full reproducibility?

Yes

Is the study design appropriate and is the work technically sound?

Yes

Are the conclusions drawn adequately supported by the results?

Yes

Are sufficient details of methods and analysis provided to allow replication by others?

Partly

Reviewer Expertise:

Gynecology,Nursing education.Clinical nursing AND teaching,PEADS,ICU NURSING

We confirm that we have read this submission and believe that we have an appropriate level of expertise to confirm that it is of an acceptable scientific standard.

# Reviewer report: Reviewer response for version 2

**DOI:** 10.5256/f1000research.203296.r497888

**Published:** 2026-07-11

**Authors:** Omar Al Omari

**Affiliations:** 1 Sultan Qaboos University, Muscat, Oman

The authors have adequately answered and responded to most of my comments. Therefore, I would like to amend my previous recommendation and state that I find this article suitable for publication without any further amendments.

Is the work clearly and accurately presented and does it cite the current literature?

Yes

If applicable, is the statistical analysis and its interpretation appropriate?

Yes

Are all the source data underlying the results available to ensure full reproducibility?

No

Is the study design appropriate and is the work technically sound?

Yes

Are the conclusions drawn adequately supported by the results?

Yes

Are sufficient details of methods and analysis provided to allow replication by others?

Partly

Reviewer Expertise:

Nursing

I confirm that I have read this submission and believe that I have an appropriate level of expertise to confirm that it is of an acceptable scientific standard.

# Response

**Published:** 2026-07-11

**Authors:** Dr. Richard Mottershead

**Affiliations:** College of Health Sciences, University of Sharjah, Sharjah, Sharjah, United Arab Emirates

On behalf of the research team, I would like to thank Prof. Omar Al Omari for his expert review and suggestions which we have assimilated into the newest version. 

 Kindest regards 

 Dr. Richard Mottershead

# Reviewer report: Reviewer response for version 1

**DOI:** 10.5256/f1000research.197572.r476700

**Published:** 2026-05-26

**Authors:** Omar Al Omari

**Affiliations:** 1 Sultan Qaboos University, Muscat, Oman

The researchers discuss an important topic: Peer Tutoring as a Student-Centered Pedagogy in Nursing Research Education: Effects on Knowledge Acquisition and Academic Self-Confidence.

**Overall Comment**

 The manuscript requires substantial English-language editing to improve grammar, clarity, sentence structure, and academic tone. For example, the following sentences require correction: “I addition, students in the experimental…” and “Both experimental and control group received standard faculty-led lectures…”.

**Title**

 The title needs to be revised because capitalization and hyphenation are inconsistent. Please revise the title as follows:

 Peer Tutoring as a Student-Centered Pedagogy in Nursing Research Education: Effects on Knowledge Acquisition and Academic Self-Confidence

**Abstract**

 The researchers stated: “The aim of this study evaluated the effectiveness of peer tutoring…”. Please revise this sentence because “the aim” cannot “evaluated.” A better version would be: “This study aimed to evaluate the effectiveness of peer tutoring…”

 The researchers stated: “A quasi-experimental post-test control group design was conducted…”. Please revise this sentence because a design is not “conducted”; rather, a study is conducted using a design. This may be rewritten as: “A quasi-experimental post-test-only control group study was conducted at…”

 The researchers stated: “Students exposed to peer tutoring achieved significantly higher knowledge scores…”. This wording is too strong for a non-randomized post-test-only design. The language should be softened. For example: “Students in the peer-tutoring group had significantly higher post-intervention knowledge scores…”

 Similarly, in the conclusion, the researchers stated: “Peer tutoring is an effective strategy for enhancing knowledge acquisition and academic self-confidence in nursing research education.” Please soften this conclusion to match the study design. For example: “Peer tutoring may be a promising strategy for enhancing knowledge acquisition and academic self-confidence in nursing research education.”

**Introduction**

 In the first and second paragraphs, the researchers repeated the definition of peer tutoring almost verbatim. For example, they stated: “Peer tutoring is a collaborative learning strategy in which students teach and support one another, fostering academic…”. Please delete one of the two definitions and retain a single, concise definition.

 The researchers stated: “Evidence from nursing education literature indicates that peer tutoring contributes positively to students and non-students academic achievement, satisfaction, and sense of belonging.” The phrase “non-students” is unclear. Please clarify what is meant by this term.

 The researchers stated: “To date, no published studies have systematically examined the impact of peer tutoring on learning outcomes in undergraduate nursing research courses in Oman or the wider Gulf region.” This statement should not depend only on the researchers’ knowledge. Please revise it to indicate that a structured literature search was conducted

**Methods**

 The researchers stated: “A quasi-experimental post-test control group design was employed to examine the…”. The design should be described more precisely as: “A quasi-experimental post-test-only non-equivalent control group design was used.”

 This description is more appropriate because no pre-test was conducted and the groups were not equivalent at baseline.

 The researchers stated: “This design was selected to allow comparison between students exposed to the peer tutoring intervention…”. Although this is correct, the researchers should explicitly acknowledge that the absence of pre-test measures limits the ability to establish baseline equivalence for knowledge and self-confidence.

 The researchers stated: “Approximately 200 students were registered across morning and evening sections of the course. A convenience sampling technique was used to recruit participants from both sections.” However, the researchers did not explain how 137 of the 200 registered students were included or whether non-participants differed from participants. Please add a paragraph reporting the number of students approached, eligible, consented, excluded, and included in the final analysis.

 Based on the manuscript, students were allocated to the control and experimental groups according to course timing: the experimental group was from the morning section and the control group from the evening section. It is unclear whether the timing of these sections was randomized. Morning and evening sections may differ in age, employment status, motivation, academic performance, and other characteristics. Because allocation was based on course sections rather than randomization, section-related confounding should be addressed analytically and discussed as a major limitation.

 In the sample size section, the reported effect size, ƒ² = 0.30, is inconsistent with a two-group t-test. The authors should report Cohen’s d instead.

 The researchers stated: “Students who were absent during scheduled peer-tutoring sessions were excluded from data collection.” Excluding absent students after allocation may introduce attrition bias and overestimate the effectiveness of the intervention. Please report the number of students excluded due to absence and compare included versus excluded students where possible.

 The researchers stated: “An open invitation to serve as a peer tutor was extended to the students in the experimental…”. Selecting tutors only from the experimental group may introduce intervention-intensity bias and tutor advantage. Please clearly state whether peer tutors were excluded from the final analysis. If they were included, please report outcomes separately for peer tutors and tutees.

 The researchers stated: “Faculty members evaluated volunteer tutors using a standardized microteaching Criteria Checklist assessing content accuracy, clarity, organization, engagement strategies…”. The checklist was not described in sufficient detail. Please describe the checklist domains, scoring system, inter-rater reliability, selection threshold, and whether assessors were trained.

 The researchers stated: “Students in the control group completed a self-reported questionnaire and academic self-confidence only.” This sentence suggests that the control group did not complete the knowledge assessment. Please clarify whether the control group completed both the knowledge test and the academic self-confidence scale.

 The researchers stated: “Knowledge of nursing research concepts was measured using 10 faculty-develo…”. Please provide evidence regarding the validity and reliability of this faculty-developed instrument.

 Please also report Cronbach’s alpha for the Academic Self-Confidence Scale.

 Independent samples t-tests were used for preliminary between-group comparisons. Given the observed baseline differences in age and CGPA, supplementary adjusted analyses, such as ANCOVA or regression, should be considered to examine whether group differences remain significant after accounting for these variables. If the authors are unable to conduct these analyses at this stage, this should be clearly acknowledged as a limitation. For example: “Although independent samples t-tests were used to compare outcomes between groups, baseline differences in age and CGPA may have influenced the findings. Future studies should consider adjusted analyses or randomized allocation to reduce the potential effect of confounding.”

**Ethical Considerations**

 Because the intervention occurred within a course, the researchers should explain how coercion was minimized and whether data collectors were independent from course instructors. Please also clarify whether consent was obtained by a researcher who was not involved in grading and whether instructors were blinded to students’ participation status during assessment

**Results and Tables**

 In Table 1, the authors mention “Chi-square/ANOVA.” However, it is unclear where ANOVA was used. If this column only reports statistical significance, please replace the column title with p-value.

 In Table 1, percentages appear to have been calculated using the total sample denominator rather than group-specific denominators. For example, male participants in the experimental group are shown as 5.8%, although 8/70 = 11.4%. Please recalculate all percentages using group-specific denominators.

 The researchers stated: “None of the participants reported low perceived usefulness. These findings indicate a consistently positive perception of peer tutoring among students exposed to the intervention.” This is an important finding and should be clearly reported and considered in the Discussion.

 The researchers stated: “Additional analyses examining associations between demographic variables and outcome measures revealed that higher CGPA, older age, and off-campus residence were significantly associated with higher knowledge and academic self-confidence scores (p < .05).” Please add a table showing these associations.

**Discussion**

 The researchers stated: “A notable contribution of this study is the significant enhancement of academic self-confidence among students who engaged in peer tutoring.” The word “enhancement” implies that baseline data were collected and compared with post-intervention data. However, no baseline self-confidence was measured. I recommend revising this sentence to state that there was a significant difference between the two groups rather than an enhancement over time.

 The researchers stated: “Nevertheless, despite these baseline differences, the peer tutoring intervention was associated with significant improvement…”. This is the central methodological issue in the study. Therefore, the sentence should be rewritten as: “Because age and CGPA differed significantly between groups, unadjusted between-group differences should be interpreted cautiously.”

 The researchers stated: “At the institutional level, the adoption of peer tutoring programs represents a cost-effective and sustainable strategy…”. Cost-effectiveness was not measured in this study. Therefore, this sentence should be softened. For example: “At the institutional level, peer tutoring may represent a feasible and potentially sustainable strategy; however, future studies are needed to examine its cost-effectiveness.”

 The researchers stated: “Baseline differences in age and CGPA between the experimental and control groups may have influenced outcomes, although statistical analyses were conducted to account for these differences.” This statement is inaccurate because the results report independent t-tests and correlations only; no adjusted analysis is presented. Please revise this statement and acknowledge this as a serious limitation of the study.

**Limitations**

 The limitation section should be strengthened by explicitly discussing the following issues: absence of randomization, absence of pre-test measures, possible contamination, ceiling effects, tutor/tutee heterogeneity, self-report bias, lack of blinding, baseline differences in age and CGPA, and lack of adjusted analyses

Is the work clearly and accurately presented and does it cite the current literature?

Yes

If applicable, is the statistical analysis and its interpretation appropriate?

Yes

Are all the source data underlying the results available to ensure full reproducibility?

No

Is the study design appropriate and is the work technically sound?

Yes

Are the conclusions drawn adequately supported by the results?

Yes

Are sufficient details of methods and analysis provided to allow replication by others?

Partly

Reviewer Expertise:

Nursing

I confirm that I have read this submission and believe that I have an appropriate level of expertise to confirm that it is of an acceptable scientific standard, however I have significant reservations, as outlined above.

# Response

**Published:** 2026-06-04

**Authors:** Dr. Richard Mottershead

**Affiliations:** College of Health Sciences, University of Sharjah, Sharjah, Sharjah, United Arab Emirates

We thank the reviewer for their detailed and constructive comments. In response, the manuscript has undergone comprehensive language editing to improve grammar, clarity, sentence structure, readability, and academic tone throughout. The title has been revised to ensure consistent capitalization and hyphenation.

 The abstract has been updated to improve methodological accuracy. The aim statement has been corrected, the study design has been more precisely described as a quasi-experimental post-test-only control group study, and the language used to describe the findings and conclusions has been moderated to better reflect the limitations of a non-randomized design.

 The introduction has been strengthened through the removal of repetitive definitions, clarification of ambiguous terminology, and revision of statements regarding the absence of regional studies to reflect a structured review of the available literature.

 The methods section has been substantially revised. The study design is now described as a quasi-experimental post-test-only non-equivalent control group design. Additional information has been added regarding participant recruitment, eligibility, consent, exclusions, and final sample composition. Clarification has also been provided regarding group allocation, which was based on existing course sections rather than randomization, and this potential source of confounding has been acknowledged as a limitation. The sample size calculation has been revised using Cohen’s d. Further clarification has been added regarding peer tutor selection, with confirmation that peer tutors were excluded from the final analysis.

 Additional detail has been included regarding instrument development, validity, reliability, and internal consistency. The Academic Self-Confidence Scale reliability coefficient for the current sample has been reported, and limitations regarding the faculty-developed knowledge assessment have been acknowledged.

 The ethical considerations section has been expanded to explain how coercion was minimized, how voluntary participation was ensured, and how confidentiality was maintained. Statistical reporting and table presentation have been corrected, including recalculation of percentages, revision of table headings, and inclusion of additional analyses requested by the reviewer.

 The discussion has been revised to avoid causal interpretations and to ensure consistency with the study design. Statements regarding effectiveness, improvement, sustainability, and institutional impact have been moderated. Finally, the limitations section has been substantially expanded to acknowledge the absence of randomization, lack of baseline measures, potential contamination, self-report bias, lack of blinding, baseline group differences, attrition bias, tutor–tutee heterogeneity, ceiling effects, and the absence of adjusted analyses.

# Reviewer report: Reviewer response for version 1

**DOI:** 10.5256/f1000research.197572.r476702

**Published:** 2026-04-30

**Authors:** Rajeswari Singaravelu

**Affiliations:** 1 Sri Ramachandra Institute of Higher Education and Research (Deemed to be University), Chennai, Tamil Nadu, India

In the abstract under methods, for statistical analysis, - association was mentioned, but no relevant statistical test and its findings were provided

 Why was a post-test only design chosen to assess the intervention effectiveness- why not pretest – posttest?

 Groups were based on morning vs evening sessions, not randomized., No explanation was provided for whether both groups are similar in terms of baseline.

 What sampling technique was used to choose the participants?

 When was the outcome assessed?

 Sample size- error effect size reporting in sample size section-effect size (ƒ² = 0.30)?

 Knowledge questionnaire – was reliability performed, how the knowledge score was interpreted?

 Tables and figure are not properly presented

 Why adjusted regression analysis not performed, as there was a baseline difference in the age and CGPA?

Is the work clearly and accurately presented and does it cite the current literature?

Yes

If applicable, is the statistical analysis and its interpretation appropriate?

Yes

Are all the source data underlying the results available to ensure full reproducibility?

Yes

Is the study design appropriate and is the work technically sound?

Yes

Are the conclusions drawn adequately supported by the results?

Yes

Are sufficient details of methods and analysis provided to allow replication by others?

Yes

Reviewer Expertise:

Womens health

I confirm that I have read this submission and believe that I have an appropriate level of expertise to confirm that it is of an acceptable scientific standard.
